# Development and Characterization of a Biodegradable Radiopaque PLA/Gd_2_O_3_ Filament for Bone-Equivalent Phantom Produced via Fused Filament Fabrication

**DOI:** 10.3390/polym17233193

**Published:** 2025-11-30

**Authors:** Özkan Özmen, Sena Dursun

**Affiliations:** 1 Department of Industrial Design Engineering, Faculty of Engineering, Erciyes University, 38039 Kayseri, Türkiye; 2 Graduate School of Natural and Applied Sciences, Department of Industrial Design Engineering, Erciyes University, 38039 Kayseri, Türkiye; sena3dursun@gmail.com

**Keywords:** additive manufacturing, fused filament fabrication, radiopaque filament, computed tomography, X-ray imaging, bone-equivalent phantom

## Abstract

Additive manufacturing (AM) has rapidly evolved due to its design flexibility, ability to enable personalized fabrication, and reduced material waste. In the medical field, fused filament fabrication (FFF) facilitates the production of individualized anatomical models for surgical preparation, education, medical imaging, and calibration. However, the lack of filaments with X-ray attenuation similar to that of biological hard tissues limits their use in radiological imaging. To address this limitation, a radiopaque filament was developed by incorporating gadolinium oxide (Gd_2_O_3_) into a biodegradable poly(lactic acid) (PLA) matrix at 1, 3, and 5 wt.%. Thermal and rheological properties were characterized using differential scanning calorimetry (DSC), thermogravimetric analysis (TGA), and melt flow index (MFI) analyses, revealing minor variations that did not affect printability under standard FFF conditions (200 °C nozzle, 60 °C build plate, 0.12 mm layer height). Microstructural analysis via field emission scanning electron microscopy (FESEM), energy-dispersive X-ray spectroscopy (EDX), elemental mapping, and micro-computed tomography (micro-CT) confirmed homogeneous Gd_2_O_3_ dispersion without nozzle blockage. Radiopacity was evaluated using gyroid infill cubes, and increasing Gd_2_O_3_ content enhanced X-ray attenuation, with 3 wt.% Gd_2_O_3_ reaching Hounsfield Unit (HU) values comparable to cortical bone. Finally, the L1 vertebra phantom fabricated from the 3 wt.% Gd_2_O_3_ filament exhibited mean HU values of approximately +200 to +250 HU at 50% infill density (trabecular bone region) and around +1000 HU at 100% infill density (cortical bone region), demonstrating the filament’s potential for producing cost-effective, radiopaque, and biodegradable phantoms for computed tomography (CT) imaging.

## 1. Introduction

Additive Manufacturing (AM) has advanced rapidly in recent years, becoming an innovative production technology now even used in households, thanks to its numerous benefits over traditional methods—such as design flexibility, the capacity for customized production, and the potential to reduce material waste. Among various AM techniques, fused filament fabrication (FFF)-based 3D printers not only offer a wide range of applications—from surgical planning and anatomical education to patient-specific orthosis and prosthesis production and dental applications—due to their ability to provide personalized medical solutions, but also hold significant potential in the field of radiology. Medical phantom models used for imaging calibration, dosimetry, training, and image optimization can be additively manufactured via FFF technology to yield anatomically realistic forms with fully customizable radiological properties that replicate specific tissue-equivalent behaviors. The diversity of these phantoms is as broad as the imaging techniques and applications in which they are used, encompassing a wide spectrum ranging from simple models that represent basic anatomy to highly detailed anatomical replicas [1,2,3,4,5].

Materials chosen for phantom fabrication need to closely replicate how biological tissues interact with X-rays, which is especially important for computed tomography (CT) imaging. In CT imaging, the ability of tissues to absorb X-rays is quantified in Hounsfield Units (HU). Low-density biological tissues, such as air-filled lungs, have negative HU values between −700 and −1000, while high-density bones, such as trabecular and cortical bone, range from +200 to +1300 HU. Researchers have developed various strategies for FFF-based 3D printers to realistically produce the wide HU scale in anthropomorphic phantoms. One of these strategies involves optimizing printing parameters to replicate soft tissues within the low HU range using commercial filaments. While commercial poly(lactic acid) (PLA) filament has been shown to achieve radiological conformity with human thyroid tissue [6], printing parameters—such as infill density [7], the combined effect of infill density and infill pattern [8], extrusion rate, and controlled underfilling [9,10,11,12]—have been shown to play a decisive role in determining HU values. In particular, by employing low infill ratios, values as low as −900 HU have been achieved, enabling the simulation of low-density tissues such as lung, breast, and adipose tissue.

Radiopacity in polymeric materials can be achieved by dispersing fillers characterized by high X-ray attenuation coefficients within the polymer matrix, enabling the resulting composite to exhibit radiodensity values comparable to those of biological tissues. Commonly used additives are high-density powders containing elements with large atomic numbers and K-edge binding energies that match the photon energy range used in medical imaging systems [13]. Commercial PLA filaments containing various additives (Cu, Al, brass, and bovine bone), as well as wood-filled ABS, HIPS, PETG, PVA, TPU, and TPE materials, have been evaluated at different infill ratios; at 100% infill, the PLA+Cu filament (containing 25–30 wt.% copper) achieved a radiodensity of approximately 330 HU [14]. In addition, studies conducted with commercially available PLA+ filaments containing metal powders, such as bronze, copper, aluminum, iron, and tungsten, have shown that varying infill and pattern parameters can achieve CT densities reaching up to 3061 HU, particularly with tungsten-filled filaments [15]. Studies conducted using PLA-based filaments containing bronze (Bronzefill) and copper (Copperfill) powders have also achieved high HU values consistent with cortical bone equivalence [16]. Similarly, by using optimized printing parameters with a PLA-Fe filament containing 45 wt.% Fe, CT densities of up to 2449 HU were achieved, successfully achieving bone tissue equivalence [17]. Lattice-printed structures developed through layered combinations of PLA and PLA-Fe filaments have provided a cost-effective manufacturing approach in FFF systems, enabling the attainment of HU ranges corresponding to cortical and trabecular bone tissues [18]. Using a dual-extrusion FFF system with commercial PLA/PLA-StoneFil filaments, a radiodensity of approximately 630 HU was achieved, providing a representative equivalence to bone tissue [19]. In another study performed using PLA+Cu and ABS filaments, a trabecular bone equivalence was achieved with a radiodensity of 428 HU at a 90% infill ratio [20]. A radiodensity of approximately 900 HU was achieved using a PLA/PLA–50 wt.% stone powder composite filament [21]. Similarly, PLA and PLA-StoneFil filaments, using a custom dual-motor extruder, achieved voxel-level control of HU values within the range of 300–800 HU [22]. Furthermore, it has been reported that standard commercial filaments such as PETG and CPE reached radiodensities of 167.76 HU and 160.06 HU, respectively; however, they were only comparable to spongy (trabecular) bone tissue [23]. A recent study reported that commercial filaments such as PLA, granite-filled PLA, PETG, and ABS provided sufficient radiopacity for the representation of soft tissue and lung at varying infill ratios; however, the available filaments were found to be inadequate for bone tissue representation [24].

Although certain commercial filaments have achieved relatively high HU ranges, researchers have pursued the development of novel, customized composite filaments to enable a more realistic representation of high-density tissues such as cortical bone. A new composite filament formulated by incorporating high-purity bismuth powder into an ABS matrix achieved a mean radiodensity of approximately 738 HU, corresponding to cortical bone equivalence, using the FFF technique, and was successfully employed in the fabrication of a patient-specific head phantom [25]. Similarly, CT densities ranging from 1438 to 2603 HU were achieved in ABS-based filaments containing up to 5% Bi_2_O_3_ [26], or up to 10% BaSO_4_; likewise, values between 1607 and 2624 HU were reported for XCT-A, XCT-B, and XCT-C filaments incorporating BaSO_4_ and CaCO_3_, thereby enabling the representation of not only trabecular and cortical bone tissues but also high-density structures such as dentin and enamel [27]. Furthermore, in a recent study, a filament developed by incorporating 12 wt.% Bi_2_O_2_CO_3_ into an ABS matrix achieved a radiodensity of approximately 1910 HU, enabling the successful fabrication of a pediatric head–neck phantom [28]. CT densities ranging from 100 to 3000 HU were obtained using seven different custom filament formulations. Composite filaments were produced by incorporating various fillers into ABS and PLA matrices: ABS-CSH (80%ABS/20%cesium sulfate hydrate), ABS-CE (80%ABS/20%cement), ABS-S (80%ABS/20%silica gel), ABS-SB20 (80%ABS/20%barium sulfate), ABS-SB10 (90%ABS/10%barium sulfate), ABS-G (80%ABS/20%Gypsum), and PLA-SSB (90%StoneFil/10%barium sulfate) [29]. They demonstrated the capability to represent a wide spectrum of tissues, ranging from soft tissue to dense cortical bone.

In studies conducted with PLA-based filaments, bone-equivalent HU values were achieved using granules containing 1.5 wt.% Bi_2_O_3_ [30]; PLA/PBAT filaments containing 2–10 wt.% BaSO_4_ achieved radiodensity values ranging from 371 to 1013 HU, with 8 wt.% BaSO_4_ in particular providing densities corresponding to trabecular bone [31]. A composite filament developed by incorporating 6 wt.% zirconia into the PLA matrix achieved an intensity of 184 HU, indicating that further increase in zirconia content may render the material suitable for the fabrication of cortical bone–equivalent phantoms [32]. In a recent study, a novel radiopaque filament was developed by incorporating 3, 5, 10 wt.% Sb_2_O_3_ into the PLA matrix, achieving radiopacity values up to 2546 HU. This filament with a 5 wt.% loading successfully reproduces the HU range corresponding to cortical bone tissue [33].

In the current literature, researchers have been prompted to explore novel filler systems due to the limited radiopacity of commercial filaments, aiming to enhance performance in both imaging compatibility and structural integrity. Gadolinium oxide (Gd_2_O_3_), one of the rare-earth oxides, exhibits a high X-ray absorption capacity owing to its K-edge located around 50 keV, according to the NIST database. Furthermore, according to the same database, at clinical CT energies around 120 keV, Gd_2_O_3_ exhibits higher attenuation performance than the commonly used radiopacifying agents Sb_2_O_3_ and BaSO_4_, while remaining lower than Bi_2_O_3_ [34,35]. Given its density and linear attenuation coefficient, it occupies less volume within the polymer matrix to achieve the required radiopacity for CT imaging. Moreover, its excellent thermal stability, chemical inertness, and neutral white color provide reliability and ease of use during both fabrication and application processes. When these distinctive features are considered together with the demand in the literature for fillers that provide high attenuation coefficients within the diagnostic CT energy range, Gd_2_O_3_ emerges as a strong candidate additive for the development of highly radiopaque filaments suitable for FFF technology and CT-based applications.

In this study, the radiopacity properties of PLA/Gd_2_O_3_ composite filaments produced by incorporating 1 wt.%, 3 wt.%, and 5 wt.% Gd_2_O_3_ into a biodegradable PLA matrix was investigated. The HU values were optimized by employing a gyroid infill architecture, while the thermal and rheological behaviors were analyzed via differential scanning calorimetry (DSC), thermogravimetric analysis (TGA), and melt flow index (MFI) testing. The dispersion of Gd_2_O_3_ was evaluated using field emission scanning electron microscopy (FESEM), energy-dispersive X-ray spectroscopy (EDX), and micro-computed tomography (micro-CT), and the radiological compatibility of the developed filaments was validated by FFF and CT evaluation of a patient-specific L1 vertebra phantom. To the best of our knowledge, this is the first study to develop and characterize PLA/Gd_2_O_3_ filaments specifically tailored for FFF-based CT phantoms, to exploit a gyroid infill architecture to tune their HU values into the bone-equivalent range, and to demonstrate a patient-specific L1 vertebra application using these filaments. For clarity, the filaments were coded so that, for example, PLA/5Gd_2_O_3_ denotes the sample containing 5 wt.% Gd_2_O_3_.

## 2. Materials and Methods

### 2.1. Materials

Commercial Gd_2_O_3_ was supplied by NanoGrafi (Ankara, Türkiye) (CAS No: 12064-62-9) with a purity of 99.99%. The material exhibited a density of 7.41 g cm^−3^ and was supplied with an average particle size of approximately 325 mesh. Luminy^®^ LX175 PLA pellets (TotalEnergies Corbion, Gorinchem, The Netherlands) were used as the base polymer in this study. According to the manufacturer’s product data sheet, this high-viscosity amorphous PLA has a density of 1.24 g cm^−3^ and a melt flow index of 6 g/10 min measured at 210 °C under a 2.16 kg load. It exhibits a glass transition temperature of around 60 °C and a melting temperature of approximately 155 °C, with typical mechanical properties including a tensile modulus of about 3.5 GPa, a tensile strength of roughly 45 MPa, and an elongation at break below 5%.

### 2.2. Material Processing

#### Masterbatch Preparation and Filament Fabrication

**Figure 1:**
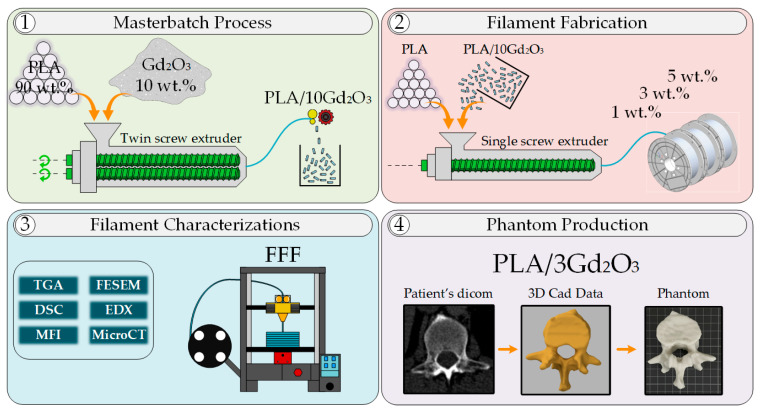
Process outline showing sequential steps of masterbatch development, filament production, characterization, and phantom creation.

Figure 1 schematically presents the methodological workflow used in this study, indicating with steps 1–4 the sequence from pellet mixing and masterbatch preparation through filament fabrication and characterization to final phantom production. The production of PLA/Gd_2_O_3_ filaments was accomplished using a two-step fabrication method. In the first stage, a co-rotating twin-screw extruder (Gülnar Makina (Kayseri, Türkiye), L/D: 48) was employed to ensure the homogeneous dispersion of Gd_2_O_3_ within the PLA matrix. PLA pellets, dried in an oven at 40 °C for 24 h, were compounded with 10 wt.% Gd_2_O_3_ using the extruder operating at 100 rpm. The applied temperature profile of 50, 190, 195, 210, 205, and 200 °C corresponded to the feed throat and three functional processing regions used in this extruder type: 50 °C for the feed throat, 190–195 °C for the feed/initial melting zone, 210–205 °C for the melting–mixing zone, and 200 °C for the metering zone prior to the die. The final zone was kept slightly cooler to prevent melt degradation. After exiting the die, the extrudate was passed through a cooling bath and subsequently granulated. The pelletizer, which is an integrated cutting unit of the twin-screw extruder (Supplementary Material, Figure S1), used a fixed 27 rpm rotating blade to produce uniformly sized pellets.

In the second stage, the PLA/10Gd_2_O_3_ masterbatch pellets were first dried at 40 °C for 24 h. Subsequently, to produce filaments containing 1 wt.%, 3 wt.%, and 5 wt.% Gd_2_O_3_, the masterbatch pellets were diluted with pure PLA to prepare the required compositions. The prepared mixtures were extruded into filaments using an industrial single-screw extruder (Filameon (Kayseri, Türkiye), L/D: 24) equipped with a laser diameter control unit and a feeding hopper. Filament fabrication was carried out using the single-screw extruder under a temperature profile of 175, 190, 200, and 210 °C, respectively. The filaments exiting the single-screw extruder were sequentially cooled in two water baths, subsequently dried with compressed air, and their diameters were measured using a laser-based system. The feed rate of the industrial extruder was regulated through feedback obtained from the laser diameter control unit. Through this system, filaments with a precision of 1.75 ± 0.05 mm were produced in accordance with commercial standards. All filaments were fabricated in quantities of approximately 1–1.5 kg (Supplementary Figure S1).

### 2.3. Characterization of Filaments and Samples

#### 2.3.1. Thermal Characterizations

TGA was carried out to investigate the thermal decomposition behavior of neat PLA, PLA/1Ga_2_O_3_, PLA/3Ga_2_O_3_, and PLA/5Ga_2_O_3_ filaments. The analyses were performed using a Hitachi-High Tech STA-7300 instrument (Hitachi High-Tech, Tokyo, Japan) under a continuous nitrogen atmosphere at a constant flow rate of 50 mL min^−1^. Each specimen was heated from ambient temperature to 800 °C at a constant ramp rate of 10 °C min^−1^.

The thermal transitions of the samples were examined using DSC. The analyses were conducted using a Mettler–Toledo DSC 1/700 instrument (Mettler-Toledo GmbH, Greifensee, Switzerland) under a nitrogen atmosphere. Each sample was subjected to a heating–cooling cycle from 25 °C to 200 °C and then back to 25 °C at a constant heating/cooling rate of 5 °C min^−1^.

#### 2.3.2. Melt Flow Index Analysis

The melt flow characteristics of the filament samples were evaluated using a Coesfeld Meltfixer LT instrument (Coesfeld Materialtest GmbH, Dortmund, Germany). During testing, a standard die measuring 8 mm in length and 2.095 mm in internal diameter was employed. All measurements were carried out at 200 °C, which reflects the extrusion temperature commonly used in FFF processes, and were performed under two different applied loads: 2.16 kg and 5 kg.

#### 2.3.3. Morphological Characterization

FESEM-EDX mapping was employed to investigate the morphological characteristics and elemental distribution of the composite samples. The analyses were performed using a ZEISS Gemini 500 instrument (Carl Zeiss Microscopy GmbH, Oberkochen, Germany). Fractured surfaces of the 3D-printed specimens (dimensions: 5 × 5 × 100 mm^3^) were examined to assess the dispersion of Gd_2_O_3_ additives within the PLA matrix. The specimens were sputter-coated with a thin Au/Pd film before imaging to enhance electrical conductivity and achieve clearer micrographs (Supplementary Figure S2).

#### 2.3.4. CT and Micro-CT Analysis

CT is a widely used imaging technique that utilizes X-rays to obtain detailed cross-sectional images of anatomical structures. In CT images, each pixel carries a numerical value that represents a specific tissue density. These values are expressed on a scale known as HU and are fundamentally based on the X-ray attenuation properties of tissues [36,37]. The HU value is determined by calculating the linear attenuation coefficient of the examined structure relative to those of pure water and air, which are taken as reference materials (Equation (1)).

(1) $$CT\;Number\;\left(HU\right)=\frac{\left(\mu -\mu_{w}\right)}{\left(\mu_{w}-\mu_{a}\right)\;}\times 1000$$

μ denotes the linear attenuation coefficient of the measured tissue; μ_w_ represents the attenuation coefficient of pure water; and μ_a_ corresponds to that of air. X-rays are attenuated to varying degrees within tissues depending on their respective attenuation coefficients. Consequently, the HU scale enables a quantitative comparison of the radiological densities of different tissues. According to the HU scale definition, the value of water is set to 0, whereas that of air is −1000.

A Toshiba Asteion system (Toshiba Medical Systems Corporation, Ōtawara, Japan) was employed to obtain the CT images. The imaging procedure was undertaken in accordance with the standard abdominal imaging protocol—consistent with the reference dataset (as detailed in Section 2.4.2)—employing a tube voltage of 120 kVp and a tube current of 100 mA. During the scan, a slice thickness and reconstruction interval of 0.5 mm were used, with an in-plane pixel spacing of 0.464 × 0.464 mm (512 × 512 matrix), and image reconstruction was carried out with the FC07 kernel (CTDIvol: 20.3 mGy). All images were exported in DICOM format after scanning.

The micro-CT samples were produced as solid cylinders (Ø 15 mm × 10 mm) employing a circular infill pattern. Each micro-CT specimen was fabricated using approximately 0.73 m of the respective filament. Micro-CT scans were conducted using a Bruker SkyScan 1272 (Bruker microCT, Kontich, Belgium) desktop micro-computed tomography system. The scans were performed in step-and-shoot mode with an angular increment of 0.6° over a rotation range of 189°. The raw projection images were acquired using an XIMEA xiRAY16 detector in 4 × 4 binning mode, with a resolution of 1224 × 1224 pixels, an isotropic pixel size of approximately 21 μm, and a bit depth of 16-bit in TIFF format. During the scanning process, various source voltages (60–80 kV), source currents (125–166 μA), exposure times (2200–6200 ms), and filters (Al, 1 mm—Cu, 0.11 mm) were used. The acquired raw images were reconstructed using NRecon software (v1.7.4.2) with the InstaRecon engine (v2.0.4.5). A Hamming filter (Alpha = 0.54), Gaussian smoothing (kernel size: 2), and ring artifact correction (level: 10–13) were applied, along with beam hardening correction when required. The resulting images were exported in BMP format.

Segmentation of the acquired micro-CT images was performed using Dragonfly 2022.2 software (Comet Technologies Canada Inc., Vancouver, BC, Canada, 2023) [38]. To prevent artifacts potentially occurring at the image edges from adversely influencing the analysis results, the raw images were restricted to a cylindrical mask with a diameter of 12 mm and a height of 8 mm before analysis. Subsequently, the images were segmented into three classes: Gd_2_O_3_, pores, and the PLA matrix. The deep learning-based segmentation model was trained using a dataset comprising 15 images for each composite filament, where the images were manually classified in conjunction with the Otsu thresholding method. The model was built on the U-Net architecture, with a depth of 5 and an initial filter count of 64. The trained deep learning model was applied to the entire dataset, completing the segmentation process. In this way, the distribution of Ga_2_O_3_ particles within the PLA matrix along approximately 0.73 m of filament length was examined for each composition.

### 2.4. Three-Dimensional Printing and Design

The samples were fabricated using a cost-efficient Ender-3 Max Neo FFF printer (Shenzhen Creality 3D Technology Co., Ltd., Shenzhen, China) featuring a 30 × 30 cm^2^ tempered-glass build plate. The printer was fitted with a 0.4 mm diameter steel nozzle. A minor modification was made by relocating the extruder directly above the hotend, effectively converting the machine into a direct-drive system. This adjustment minimized filament bending and buckling during extrusion, thereby improving overall print quality.

#### 2.4.1. Calibration Cubes

The calibration cubes and their internal structures were created using the volumetric lattice tool in the design extension module of Autodesk Fusion 360 (Version 2605.0.97, 2025) software. A gyroid pattern was selected for the trabecular bone part due to its excellent modeling performance under CT imaging [33]. The cubes were designed with dimensions of 20 × 20 × 20 mm, incorporating a gyroid lattice structure, with the lattice parameters set to a cell size of 2 mm and an outer wall thickness of 2 mm (blend distance: 0.40 mm). The G-code files required for fabrication were generated during the slicing stage using Ultimaker Cura (Version 5.10.0) open-source software [39] (Supplementary Figure S3). In Cura, the calibration cubes were configured without top and bottom layers, and the supports were disabled. They were printed using neat PLA, PLA/1Gd_2_O_3_, PLA/3Gd_2_O_3_, and PLA/5Gd_2_O_3_ filaments. Although ten calibration cubes with infill densities ranging from 10% to 100% were designed, the calibration cube with 10% infill density could not be printed. Accordingly, calibration cubes printed with infill densities ranging from 20% to 100% were utilized. After the CT scans, a 10 × 10 mm^2^ region of interest (ROI) was selected from the center of each calibration cube, and three HU measurements were recorded. The average of these measurements was used to determine the mean HU value of each calibration cube. All reconstructed CT images were visualized using a fixed window configuration (width = 300 HU, level = 2500 HU).

#### 2.4.2. L1 Vertebra Phantom Design

The dataset from The Cancer Imaging Archive (TCIA), the TCGA Kidney Renal Clear Cell Carcinoma (TCGA-KIRC) collection, specifically images from Patient ID TCGA-CW-6088, was used to create the L1 vertebra phantom. The dataset was accessed through TCIA and employed for the 3D reconstruction and modeling of the L1 vertebra [40,41]. Image segmentation was manually performed using the open-source software 3D Slicer (Version 5.8.1) [42].

**Figure 2:**
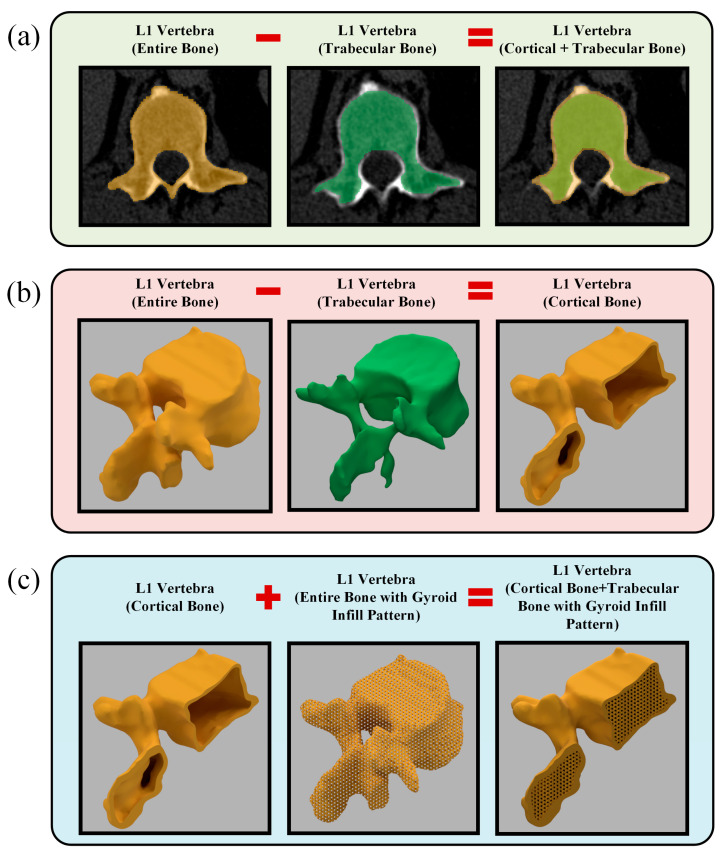
Design of the L1 vertebra phantom: (**a**) Manual segmentation of the entire L1 vertebra and its trabecular bone from CT images; (**b**) Generation of the cortical bone as a shell structure; (**c**) integration of the cortical shell with the gyroid infill pattern to reconstruct the complete L1 vertebra model.

As shown in Figure 2, the L1 vertebra phantom design process consisted of three principal stages. At the first stage, the entire L1 vertebra was segmented from the patient-derived CT dataset (Figure 2a, shown in orange). In the subsequent step, the cancellous bone region of the same vertebra was manually segmented from the CT dataset (Figure 2a, shown in green). In the final image of Figure 2a, both segmentation datasets are superimposed on the patient’s CT slice. At the second stage, the obtained segmentation data were converted into STL format and imported into Autodesk Fusion 360 software. Figure 2b presents the 3D models of the entire L1 vertebra (orange) and its trabecular region (green). At this stage, the trabecular design data of the same bone were subtracted from the entire L1 vertebra (orange) model, resulting in the isolated cortical bone model of the L1 vertebra. At the third stage, using the design module of Fusion 360, a gyroid-patterned internal infill structure with a 2 mm cell size was applied to the entire L1 vertebra (orange region in the middle of Figure 2c), based on the CT results obtained from the calibration cubes. The L1 vertebra cortical bone and the entire L1 bone with the gyroid infill pattern were merged to generate the L1 vertebra model with an internal gyroid structure. This process ensured the formation of the L1 vertebra, along with its internal trabecular and cortical bone structures, without any gaps or structural inconsistencies between the gyroid infill and the cortical shell.

The designed L1 vertebra phantom was imported into Cura in STL format, and various customized parameters were applied during slicing to generate the G-code file. Specifically, the layer height was set to 0.12 mm. This high-resolution layer thickness enabled the precise printing of the trabecular structure details. The wall line count was set to “0” to prevent Cura from automatically generating outer walls, as the outer shell geometry had already been modeled as the cortical bone in the Fusion 360 environment. In addition, the number of bottom layers was set to exceed the height of the L1 vertebra to enable manual control over the support structures. With this setting, the software treated the entire model as the first layer, generating supports only on the outer surfaces while preventing the formation of extra supports within the internal regions containing the gyroid infill structure. Thus, the necessary supports for the outer cortical shell were preserved, while the formation of unnecessary supports within the internal trabecular region was prevented. The printing speed was set to 20 mm s^−1^ for all structural elements, and the entire printing process took approximately 38 h to complete. A total of 80 g of filament was consumed during fabrication. After printing was complete, the support structures on the outer shell were carefully removed, thereby preserving the model’s surface integrity. During the CT scans, a custom stabilizing apparatus made of pure PLA was used to mimic patient positioning and prevent any movement of the phantom (Supplementary Figure S4).

## 3. Results and Discussions

### 3.1. Thermal Analysis of the Filaments

**Figure 3:**
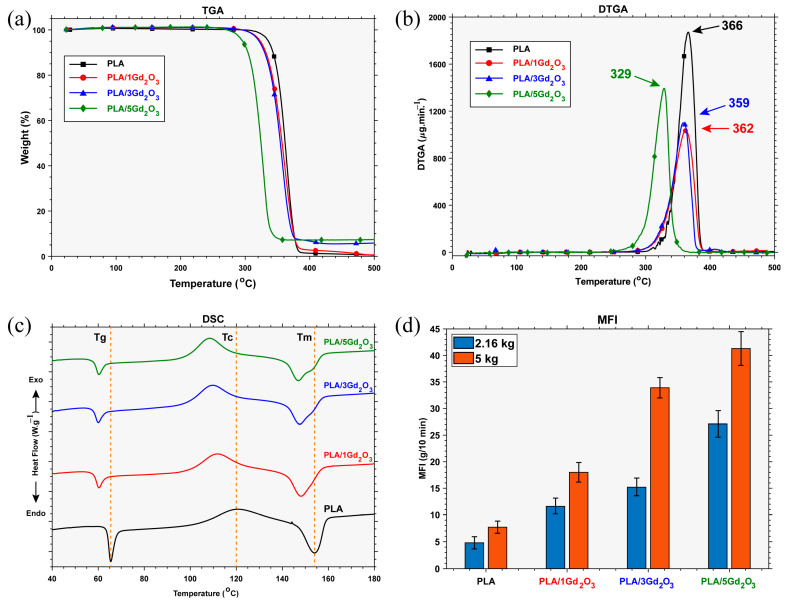
Filament characterization results: (**a**) TGA, (**b**) DTGA, (**c**) DSC, (**d**) MFI.

As shown by the TGA-DTG curves in Figure 3a,b and the numerical data in Table 1, all filaments undergo single-stage degradation. An increase in the Gd_2_O_3_ content within the PLA matrix led to an earlier onset temperature of degradation while resulting in a higher residual mass after decomposition. The observed temperature drop suggests a catalytic effect of Gd_2_O_3_ on the degradation process, accompanied by a slight reduction in the thermal stability of the PLA chains. While neat PLA degraded almost completely (0.48% residue), the PLA/5Gd_2_O_3_ composite left a residual mass of 7.40%.

This indicates that, due to its thermally stable nature, Gd_2_O_3_ remained as a residue without decomposing, and owing to its inorganic structure, it did not participate in the degradation. This observed reduction in thermal stability is consistent with reports on other radiopacifying metal oxides, such as Sb_2_O_3_, Bi_2_O_3_, and Fe_2_O_3_, within PLA composites [33,43]. However, because FFF with PLA is conducted at a nozzle (hot-end) temperature of approximately 200 °C, no significant thermal degradation occurs under these processing conditions.

Inspection of the DSC curve indicates that neat PLA exhibits a glass transition temperature (T_g_) of 65.5 °C, a cold-crystallization temperature (Tc) of 120.58 °C, and a melting temperature (Tm) of 154 °C. The presence of Gd_2_O_3_ is observed to slightly lower the glass transition temperature (T_g_) and, by acting as a nucleating agent, to initiate crystallization at lower temperatures, a behavior similar to that reported for Sb_2_O_3_-filled PLA systems [33]. Whereas neat PLA and PLA/1Gd_2_O_3_ exhibit a single melting peak, the melting region of PLA/3Gd_2_O_3_ and PLA/5Gd_2_O_3_ filaments reveals a primary melting peak accompanied by a shoulder-like feature that becomes slightly more pronounced. This observation suggests that Gd_2_O_3_ acts as a nucleating agent, thereby promoting and directing crystal growth. As the Gd_2_O_3_ content increases, its effect becomes more pronounced; at low loadings, a single melting peak is preserved, whereas at higher loadings, the crystalline structure becomes heterogeneous, and this heterogeneity is directly reflected in the melting behavior. It can be inferred that Gd_2_O_3_ incorporation brings about only insignificant deviations in the thermal transition characteristics of the composite. Although slight variations were detected, they were not significant enough to alter the extrusion temperature, since the conventional FFF processing range for pristine PLA (180–220 °C) was found to be compatible with PLA/Gd_2_O_3_ composites, in agreement with previous reports on PLA systems containing inorganic additives [30,33]. In line with this observation, the printing was carried out under standard conditions (nozzle: 200 °C; build plate: 60 °C).

### 3.2. Melt Flow Behavior of the Filaments

MFI is inversely proportional to a polymer’s melt viscosity and, consequently, directly influences key 3D-printing parameters—print speed, melt flowability, interlayer adhesion, and the quality of the final part. Examination of Figure 3d shows that, under both load conditions, MFI increases with increasing Gd_2_O_3_ content. These results show that melt flowability scales with Gd_2_O_3_ content in a straightforward yet nonlinear manner. A decrease in thermal stability led to chain scission at lower temperatures and, consequently, to a reduction in molecular weight. This reduction shortened the chains and facilitated melt flow, as evidenced by an increase in the MFI. Nevertheless, this change in melt viscosity did not produce any processing issues during 3D printing of PLA/Gd_2_O_3_ filaments under standard processing conditions.

### 3.3. Morphological Characterization of the Filaments

**Figure 4:**
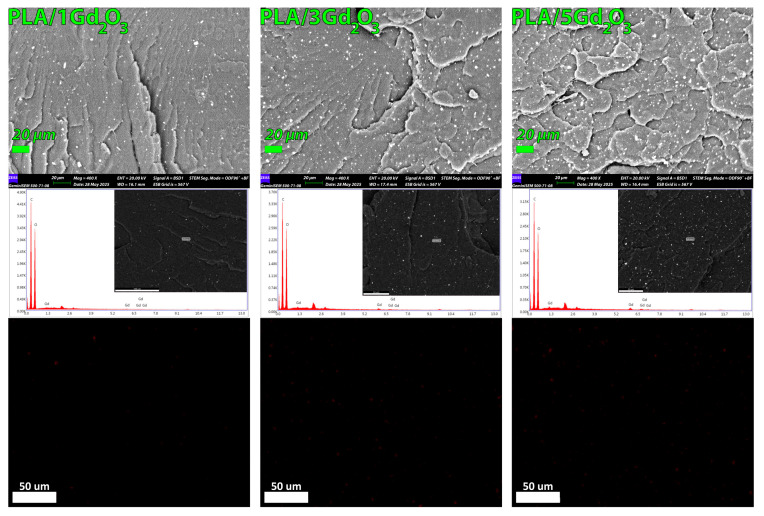
FESEM images and corresponding EDX elemental maps showing the dispersion of Gd_2_O_3_ within the PLA matrix for PLA/1Gd_2_O_3_, PLA/3Gd_2_O_3_, and PLA/5Gd_2_O_3_ filaments.

The content, quality, and spatial arrangement of Gd_2_O_3_ particles within the filaments were evaluated using FESEM imaging coupled with EDX elemental mapping of the specimens’ controlled-fracture surfaces, as shown in Figure 4. A homogeneous dispersion of Gd_2_O_3_ particles was observed across all filament compositions, indicating good compatibility with PLA over the tested concentration range. Furthermore, at the lower magnification shown in Supplementary Figure S5, no discernible particle agglomeration was observed on the examined surfaces when considering particle size.

The EDX spectrum clearly exhibits the characteristic peaks of Gd along with those of C and O, confirming the successful incorporation of Gd_2_O_3_ into the PLA matrix. With increasing Gd_2_O_3_ loading, the Gd peaks became more pronounced, confirming the concomitant increase in the detected Gd content. A similar trend was observed in the EDX elemental mapping results; no discernible particle agglomeration was detected on the surfaces of the examined specimens.

**Figure 5:**
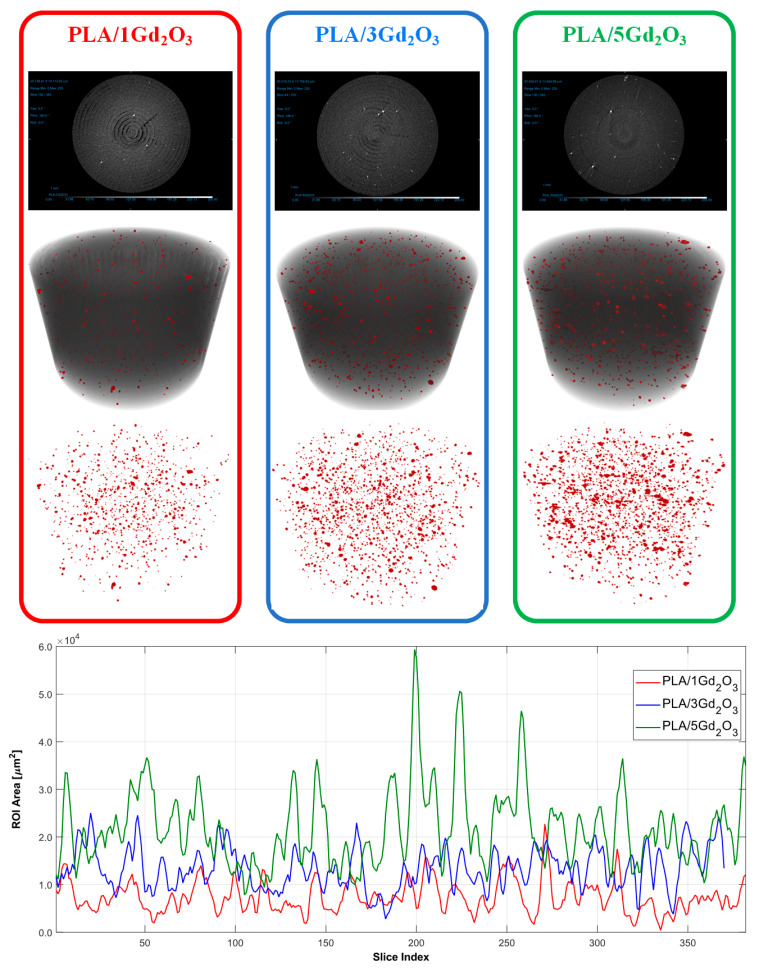
Micro-CT analysis results of PLA/1Gd_2_O_3_, PLA/3Gd_2_O_3_, and PLA/5Gd_2_O_3_ samples.

Upon examination of the raw micro-CT scans in Figure 5, it is evident that, in the PLA/1Gd_2_O_3_, PLA/3Gd_2_O_3_, and PLA/5Gd_2_O_3_ composites, the bright (white) features correspond to Gd_2_O_3_ particles, the darker gray tones to the PLA matrix, and the black regions to voids (pores). The classification results indicate a marked increase in particle density with increasing Gd_2_O_3_ loading in the PLA/Gd_2_O_3_ composites. In the PLA/1Gd_2_O_3_ sample, the filler particles were dispersed with low density and high homogeneity. However, with the increased Gd_2_O_3_ content in the PLA/3Gd_2_O_3_ sample, localized clustering tendencies were observed, although without the formation of true agglomerates. The PLA/5Gd_2_O_3_ sample, in general, maintained a homogeneous particle distribution, yet regions with partially higher particle density were discernible. Slice analyses further corroborated these observations: specimens with higher Gd_2_O_3_ loadings exhibited a larger Gd_2_O_3_-covered area within the slices. Nevertheless, across all composite filaments, neither the morphological analyses nor the printing trials revealed any agglomeration capable of causing nozzle clogging.

### 3.4. CT Characterization of the Composite Filaments

**Figure 6:**
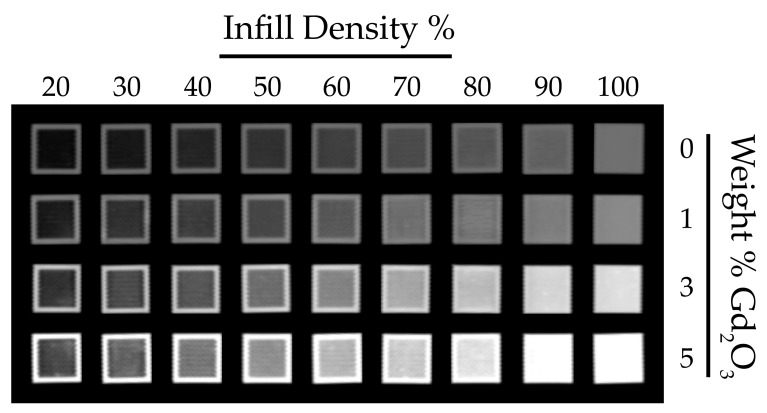
Coronal CT images of PLA/Gd_2_O_3_ calibration cubes with varying infill densities and filler contents.

**Figure 7:**
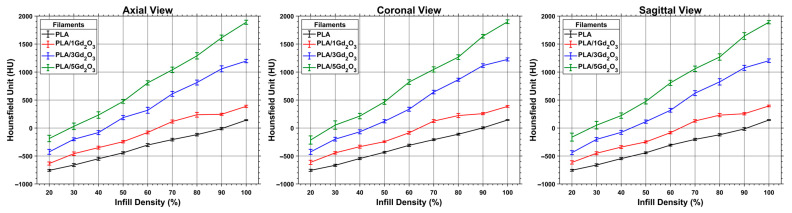
Distribution of HU values for PLA/Gd_2_O_3_ calibration cubes measured across all CT planes.

Figure 6 presents the coronal view of the calibration test cubes 3D-printed with a gyroid infill pattern. HU measurements acquired across all planes are presented in Figure 7, with the corresponding numerical values reported in Supplementary Tables S1–S3. As expected, increasing infill density reduces internal void content and consequently increases HU values. Similarly, increasing the Gd_2_O_3_ content results in a marked rise in HU values, as gadolinium’s high atomic number strongly attenuates X-rays and thereby enhances radiopacity. Furthermore, examination of HU values in the axial, coronal, and sagittal planes demonstrates that the gyroid structure yields comparable, orientation-independent measurements. Such consistency is attributable to the gyroid’s three-axis symmetry and continuous surface architecture [44].

**Figure 8:**
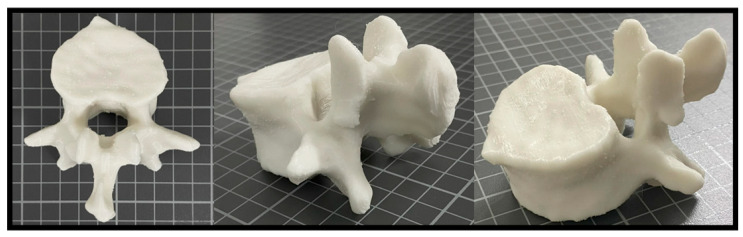
3D-printed PLA/3Gd_2_O_3_ L1 vertebra phantom shown from different perspectives.

Based on HU measurements obtained from the patient’s CT images, the PLA/3Gd_2_O_3_ filament exhibited values suitable for reproducing bone-mimicking structures and was therefore selected for phantom fabrication. Figure 8 shows the physical appearance of the L1 vertebra phantom fabricated from PLA/3Gd_2_O_3_ filament.

**Figure 9:**
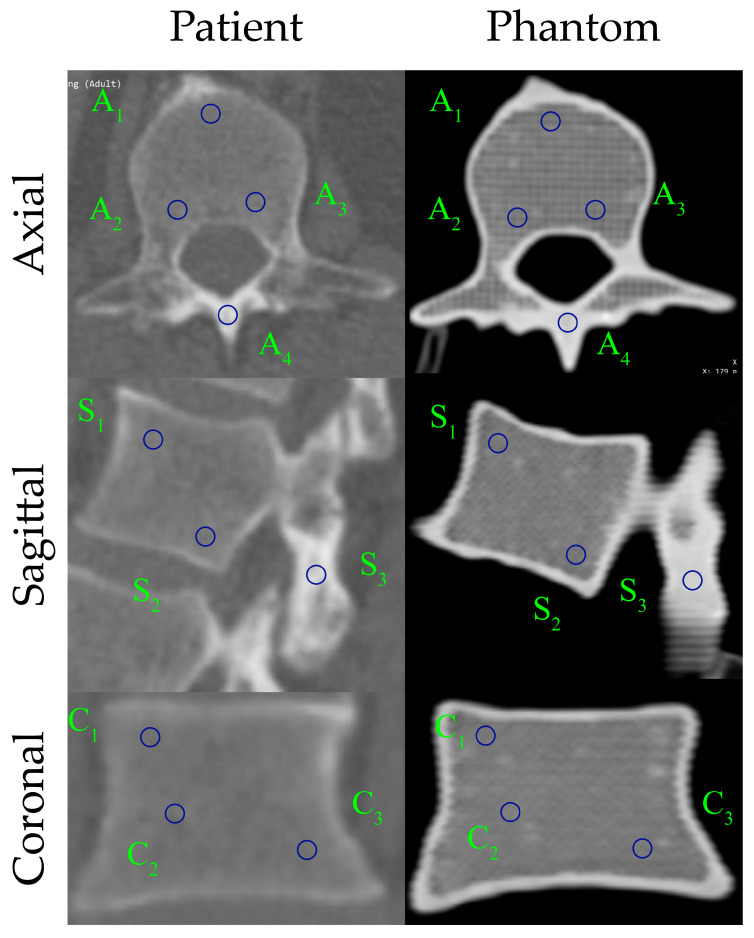
Comparison of HU distribution between the patient and the 3D-printed L1 vertebra phantom in three anatomical planes.

Comparative HU measurements for the patient’s L1 vertebra and the 3D-printed L1 vertebral phantom, obtained in the axial, sagittal, and coronal planes, are shown in Figure 9, and the corresponding numerical values, including the mean HU and standard deviation within each ROI, are presented in Table 2. Blue circles denote the ROIs used for HU measurement, corresponding to trabecular (A1–A3, S1–S2, C1–C3) and cortical (A4, S3) bone regions.

Overall, the HU-based comparisons indicate strong agreement between the patient and phantom across all planes. The L1 vertebra phantom exhibits a slight positive bias in trabecular regions, whereas cortical regions (A4, S3) closely match the patient values (≈2% error), with most patient–phantom differences remaining within ±30 HU. Concordance is highest in the sagittal plane; axial and coronal planes also show good agreement, though a few ROIs (e.g., A3 and C1–C2) display localized deviations. Standard deviations are modestly higher for the phantom in axial trabecular ROIs, while the cortical shell appears more uniform, consistent with the printed outer wall. Collectively, these findings confirm that the 3D-printed L1 phantom faithfully reproduces the patient-specific vertebra’s radiological attenuation characteristics, exhibiting only minor, manageable deviations in trabecular regions. The phantom captured HU distributions and tissue-density contrasts in three dimensions. Furthermore, the produced filament provides excellent printability, enabling precise fabrication of the vertebra’s complex geometry with high dimensional fidelity and surface integrity.

## 4. Conclusions

This study presents the development and characterization of a Gd_2_O_3_-doped PLA filament with tunable radiopacity to emulate the X-ray attenuation of hard tissues over a range of HU values. Its thermal, flow, and microstructural properties were evaluated using TGA, DSC, MFI, FESEM–EDX elemental mapping, and micro-CT.

Thermal analyses showed that the addition of Gd_2_O_3_ slightly reduced the onset degradation temperature and T_g_ and promoted earlier crystallization; however, these changes did not require any adjustment of the nozzle temperature, indicating that the composites retained good printability under conventional FFF conditions. Microstructural analyses confirmed a homogeneous dispersion of Gd_2_O_3_ particles within the PLA matrix without pronounced agglomeration.

To evaluate the applicability of the PLA/Gd_2_O_3_ filament, a patient-specific L1 vertebral model was fabricated. The resulting printed phantom closely mimicked the patient’s vertebra in terms of CT attenuation, geometry, and contrast, providing a clinically relevant model for imaging studies. However, the cortical bone layer of the L1 vertebral phantom was observed to be thicker than that of the patient’s actual vertebra, which was attributed to the manual segmentation of the patient’s CT images. Looking ahead, this limitation could be mitigated by AI-assisted automatic segmentation using separate thresholds for cortical and trabecular bone. In addition, one of the main reasons for the HU difference between the phantom and the patient’s trabecular bone is that, while trabecular bone naturally contains organic components, the phantom is composed solely of filament material and internal air voids; this discrepancy could be further reduced by multi-material 3D printing using additional filaments designed to mimic the HU response of these organic components, allowing a more realistic representation of the phantom’s internal structure.

These findings demonstrate that the newly developed biodegradable PLA-based filament containing Gd_2_O_3_ enables the attainment of bone-like radiodensities and the fabrication of cost-effective radiological calibration phantoms. Moreover, this filament is considered a promising candidate for future studies aimed at developing validation models for HU-based segmentation algorithms, optimizing imaging protocols, and producing patient-specific, detailed anatomical structures.

## Figures and Tables

**Table 1:** Thermal analysis results (TGA and DSC) of the filaments.

Sample	T_5%_ (ºC) ^a^	T_max_ (ºC) ^b^	Residue Yield (%) ^c^	T_g_ (ºC) ^d^	T_c_ (ºC) ^e^	T_m1_ (ºC) ^f^	T_m2_ (ºC) ^f^
**PLA**	336.36	366	0.48	65.5	120.58	154	
**PLA/1Gd_2_O_3_**	323.62	362	0.66	60.33	111.83	148.17	
**PLA/3Gd_2_O_3_**	322.28	359	5.83	60.08	109.83	147.58	155.08
**PLA/5Gd_2_O_3_**	296.32	329	7.40	60.33	108.50	146.92	155.08

Notes: ^a^ = Temperature at 5% weight reduction; ^b^ = the maximum degradation rate temperatures; ^c^ = residue yield at 500 °C; ^d^ = glass transition temperature; ^e^ = crystallization temperature; ^f^ = melting points.

**Table 2:** Comparative HU measurements of the L1 vertebra derived from patient and phantom CT images.

	L1 Vertebra				
	Patient (HU)			Phantom (HU)	
	Axial View				
	Mean	StdDev.		Mean	StdDev.
**A_1_**	194.94	20.92		201.11	45.50
**A_2_**	220.19	42.38		249.84	46.39
**A_3_**	206.80	35.70		273.86	57.41
**A_4_**	1057.60	76.00		1025.77	43.32
	**Sagittal View**				
	**Mean**	**StdDev.**		**Mean**	**StdDev.**
**S_1_**	240.08	34.11		226.87	32.78
**S_2_**	231.40	33.01		217.27	37.78
**S_3_**	1023.17	78.66		1032.79	71.71
	**Coronal View**				
	**Mean**	**StdDev.**		**Mean**	**StdDev.**
**C_1_**	196.31	41.89		228.65	28.66
**C_2_**	191.47	38.51		228.26	33.65
**C_3_**	191.71	33.81		197.25	39.67

## Data Availability

The original contributions presented in this study are included in the article/Supplementary Material. Further inquiries can be directed to the corresponding author.

## Supplementary Materials

The following supporting information can be downloaded at: https://www.mdpi.com/article/10.3390/polym17233193/s1, Figure S1: Twin-screw extruder for masterbatch pellet preparation and single-screw extruder for filament fabrication. Figure S2: Test specimens prepared for TGA, DSC, MFI, FESEM–EDX mapping, CT, and micro-CT analyses. Figure S3: Design and slicing workflow in Autodesk Fusion 360 and Ultimaker Cura. Figure S4: Production of the L1 vertebra phantom with PLA/3Gd_2_O_3_ and a custom holder fabricated from pure PLA to support it. Figure S5: Low-magnification (100×, scale = 100 µm) FESEM images. Table S1: Axial HU measurement results of calibration cubes with a gyroid pattern. Table S2: Coronal HU measurement results of calibration cubes with a gyroid pattern. Table S3: Sagittal HU measurement results of calibration cubes with a gyroid pattern.
