# Supplementary material for: Development and Characterization of a Biodegradable Radiopaque PLA/Gd2O3 Filament for Bone-Equivalent Phantom Produced via Fused Filament Fabrication
Source: Polymers (Basel). 2025 Nov 30;17(23):3193. doi: 10.3390/polym17233193 (PMC12693748; doi:10.3390/polym17233193)
Supplement: Supplementary file 1 [file polymers-17-03193-s001.zip › polymers-3978268-supplementary.pdf]

Development and Characterization of a Biodegradable Radiopaque PLA/Gd_2O_3 Filament for Bone-Equivalent Phantom Produced via Fused Filament Fabrication

Supporting Figures: Figure S1-S5:

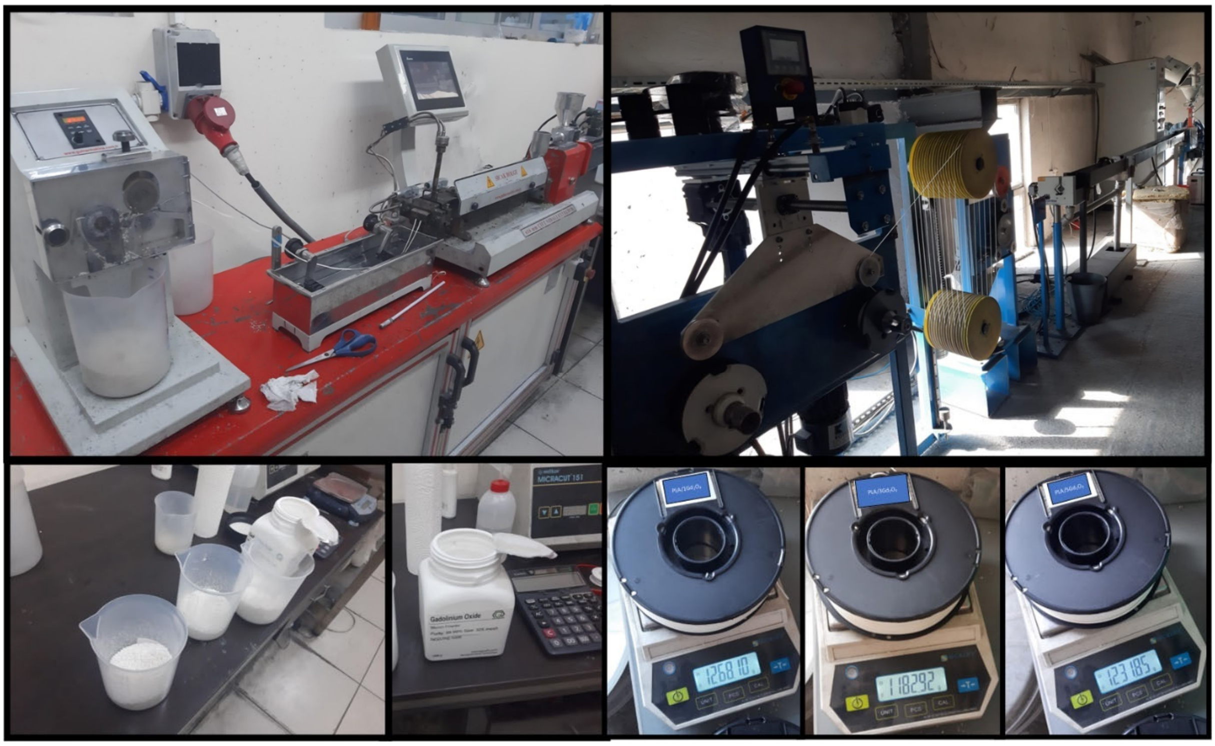

Figure S1. Twin-screw extruder for masterbatch pellet preparation and single-screw extruder for filament fabrication.

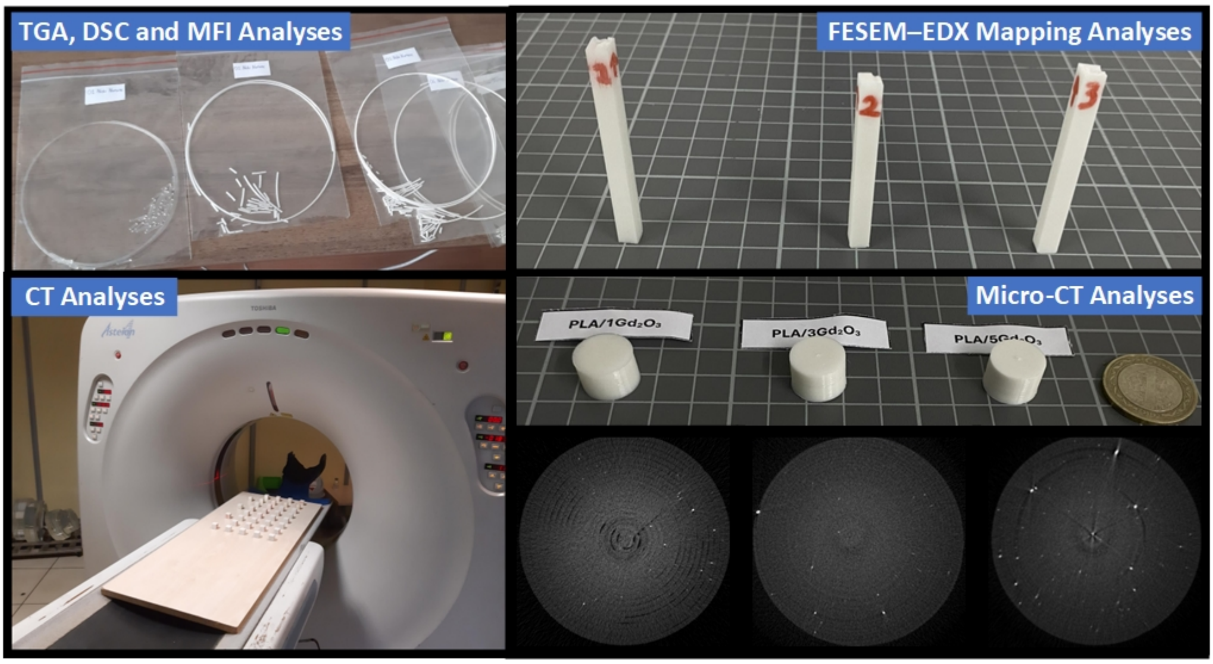

Figure S2. Test specimens prepared for TGA, DSC, MFI, FESEM–EDX mapping, CT, and micro-CT analyses.

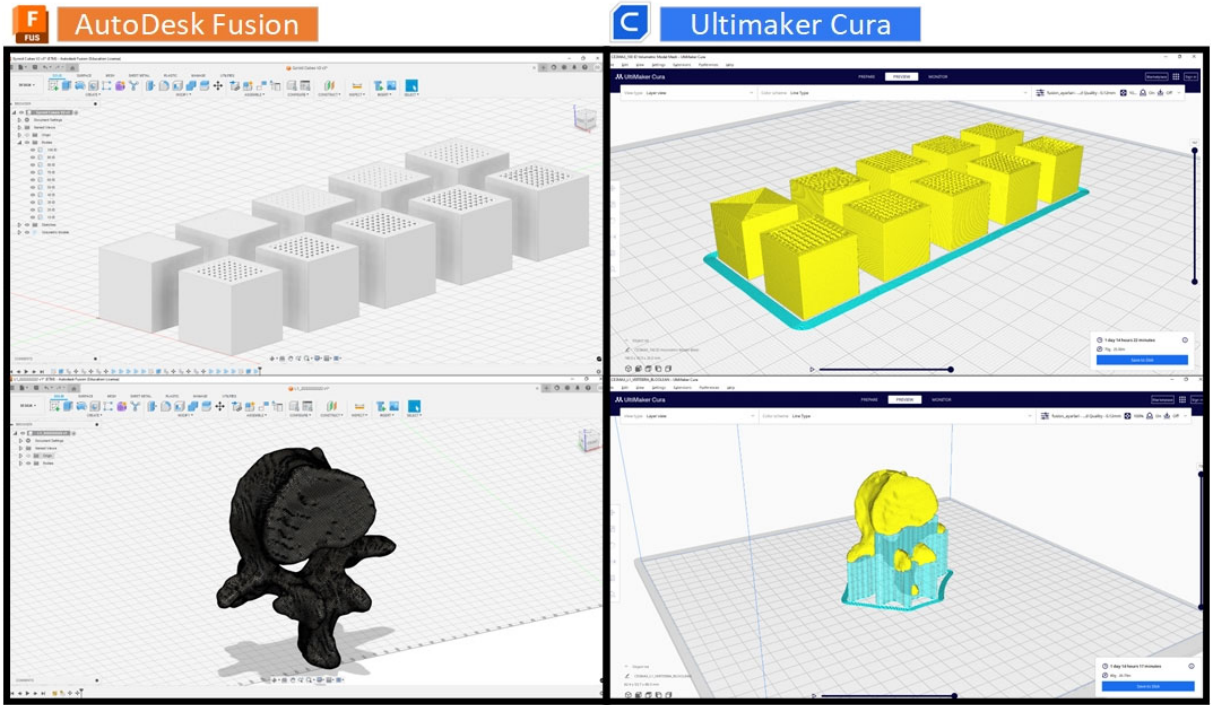

Figure S3. Design and slicing workflow in Autodesk Fusion 360 and Ultimaker Cura.

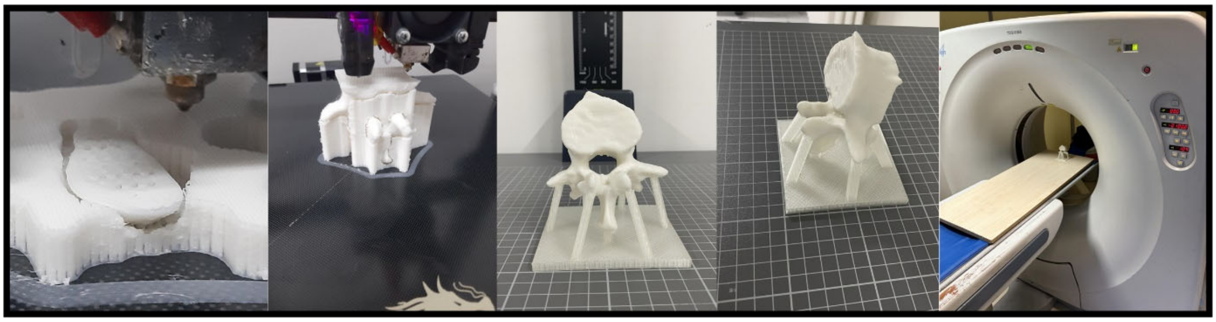

Figure S4. Production of the L1 vertebra phantom with PLA/3Gd_2O_3 and a custom holder fabricated from pure PLA to support it.

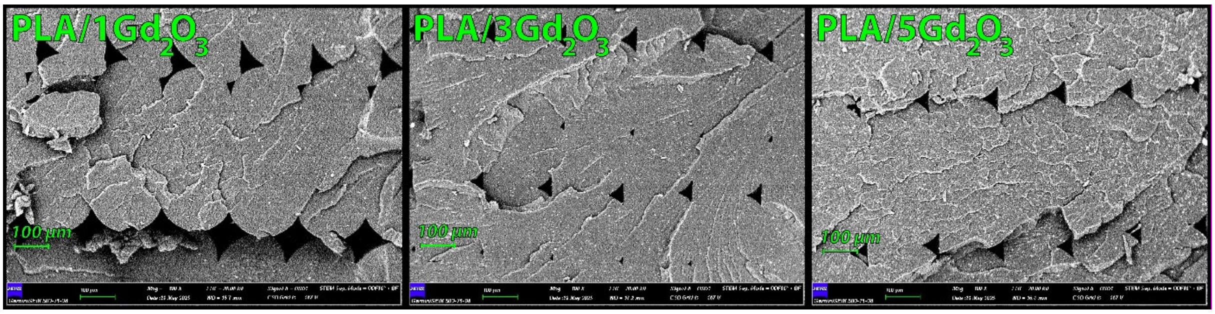

Figure S5. Low-magnification (100×, scale = 100 µm) FESEM images.

Supporting Table: Table S1 – S3

The axial, coronal, and sagittal HU data of the calibration cubes with gyroid infill pattern at varying densities are summarized in these tables. For each cube, a ROI measuring 10 × 10 mm² was selected from the central area, deliberately avoiding the outer walls. Three separate measurements were taken within this region, and their mean value was considered as the representative HU for that cube.

Table S1. Axial HU measurement results of calibration cubes with a gyroid pattern

	Pure PLA					PLA/1Gd_2O_3				
**Infill Density(%)**	**Mean**	**Median**	**SDev**	**Max**	**Min**	**Mean**	**Median**	**SDev**	**Max**	**Min**
20	-757.04	-757.00	16.15	-711.67	-798.33	-634.65	-636.83	27.89	-551.00	-697.67
30	-662.15	-662.83	25.50	-592.33	-732.67	-458.50	-457.67	30.35	-387.33	-543.33
40	-549.39	-548.83	26.89	-478.67	-612.00	-350.72	-352.17	25.00	-281.67	-406.67
50	-443.06	-442.67	20.23	-353.33	-490.33	-244.13	-243.50	21.39	-193.00	-308.00
60	-303.98	-303.50	26.13	-245.33	-377.00	-79.27	-80.17	25.41	-24.00	-150.00
70	-206.83	-206.00	23.34	-143.00	-265.00	116.18	116.67	27.54	205.67	50.67
80	-118.78	-119.83	22.33	-61.67	-173.00	237.30	247.00	42.49	318.00	69.33
90	-10.96	-8.50	20.72	32.33	-68.00	245.87	244.83	18.15	302.67	198.67
100	140.68	140.50	8.14	159.00	120.00	387.99	388.33	19.34	431.67	339.67

	$PLA/3Gd_2O_3$					$PLA/5Gd_2O_3$				
**Infill Density(%)**	**Mean**	**Median**	**SDev**	**Max**	**Min**	**Mean**	**Median**	**SDev**	**Max**	**Min**
20	-426.12	-425.67	43.83	-312.00	-530.00	-187.25	-193.33	57.32	-5.00	-315.67
30	-200.96	-201.00	25.44	-133.67	-275.00	30.63	32.17	58.04	218.00	-96.67
40	-81.56	-82.50	35.71	11.00	-178.67	232.27	231.67	57.31	414.00	101.00
50	186.32	187.33	37.99	284.00	95.33	474.69	474.33	35.96	555.00	386.33
60	317.47	310.50	53.53	513.33	216.00	806.80	809.50	37.98	890.67	704.33
70	610.92	607.17	43.78	710.00	517.00	1039.51	1036.83	48.84	1183.33	923.00
80	812.84	815.33	44.14	919.67	711.33	1289.53	1282.67	56.06	1472.33	1180.00
90	1057.58	1062.50	53.44	1142.00	939.67	1612.59	1613.83	47.52	1748.00	1505.00
100	1196.59	1195.33	30.31	1317.67	1186.67	1889.44	1889.17	36.29	1981.00	1808.67

Table S2. Coronal HU measurement results of calibration cubes with a gyroid pattern

	Pure PLA					$PLA/1Gd_2O_3$				
**Infill Density(%)**	**Mean**	**Median**	**SDev**	**Max**	**Min**	**Mean**	**Median**	**SDev**	**Max**	**Min**
20	-756.46	-757.5	19.36	-698.33	-807.67	-613.33	-616.67	36.96	-513.00	-695.33
30	-666.38	-669.33	18.91	-605.00	-714.33	-443.84	-443.83	22.08	-359.67	-500.33
40	-545.36	-545.67	18.68	-488.67	-595.00	-333.42	-335.33	27.15	-240.00	-408.00
50	-433.60	-433.33	15.18	-391.00	-478.00	-243.69	-243.67	13.43	-209.33	-276.33
60	-309.07	-308.17	19.98	-259.00	-363.33	-85.13	-84.00	24.65	-19.33	-145.00
70	-207.96	-208.00	14.82	-168.33	-250.33	124.22	122.00	24.90	231.67	56.67
80	-110.76	-109.33	17.10	-63.00	-157.67	222.78	231.33	35.14	281.00	97.33
90	3.25	3.00	17.99	51.67	-43.67	257.18	260.67	19.21	303.67	198.33
100	143.58	144.00	6.49	160.00	126.00	386.53	389.67	15.14	416.00	343.00

	$PLA/3Gd_2O_3$					$PLA/5Gd_2O_3$				
**Infill Density(%)**	**Mean**	**Median**	**SDev**	**Max**	**Min**	**Mean**	**Median**	**SDev**	**Max**	**Min**
20	-426.05	-428.50	44.87	-291.00	-533.33	-214.37	-222.83	73.68	46.33	-380.00
30	-199.09	-199.17	33.35	-102.00	-291.00	52.89	48.17	75.04	268.00	-105.00
40	-67.76	-66.67	36.04	37.67	-163.00	213.57	211.50	48.68	385.00	108.00
50	120.53	120.67	28.53	212.67	47.67	466.70	465.67	42.96	593.00	357.00
60	335.90	333.67	35.39	497.00	256.00	821.09	816.67	41.38	960.00	725.00
70	642.38	643.17	31.72	734.67	549.00	1046.80	1044.83	46.51	1214.67	936.00
80	863.11	858.67	26.89	955.67	808.00	1266.53	1267.00	40.68	1376.67	1165.67
90	1115.85	1113.50	32.04	1327.00	1055.33	1639.33	1638.33	32.56	1724.33	1541.00
100	1225.41	1226.00	27.22	1325.00	1164.33	1899.00	1901.67	33.26	2001.33	1829.33

Table S3. Sagittal HU measurement results of calibration cubes with a gyroid pattern

	Pure PLA					PLA/1Gd_2O_3				
**Infill Density(%)**	**Mean**	**Median**	**SDev**	**Max**	**Min**	**Mean**	**Median**	**SDev**	**Max**	**Min**
20	-755.36	-755.17	16.61	-704.33	-797.67	-614.68	-615.83	29.27	-519.00	-683.00
30	-661.24	-661.33	21.89	-600.33	-716.00	-450.99	-450.33	26.42	-363.67	-521.00
40	-546.41	-547.00	19.18	-487.00	-595.00	-340.66	-341.50	24.49	-280.00	-407.00
50	-442.53	-442.67	16.48	-393.00	-489.00	-248.94	-250.33	19.08	-196.00	-301.00
60	-305.80	-304.67	16.40	-265.67	-357.00	-83.38	-81.33	20.62	-35.67	-144.33
70	-202.93	-203.83	18.62	-134.33	-253.00	126.58	127.83	21.51	180.00	64.33
80	-120.41	-120.17	20.74	-71.67	-175.00	230.23	232.83	28.34	288.33	136.00
90	-16.73	-13.33	24.22	34.33	-72.00	255.88	255.33	20.92	318.00	199.67
100	143.01	144.00	7.08	158.67	125.00	394.50	395.50	15.19	436.67	356.33

	PLA/3Gd_2O_3					PLA/5Gd_2O_3				
**Infill Density(%)**	**Mean**	**Median**	**SDev**	**Max**	**Min**	**Mean**	**Median**	**SDev**	**Max**	**Min**
20	-440.35	-442.67	35.84	-316.67	-515.00	-162.55	-168.33	71.16	35.33	-323.00
30	-202.50	-203.00	32.48	-115.67	-295.00	50.89	46.33	67.28	248.00	-92.00
40	-77.14	-78.17	35.04	26.00	-158.67	222.14	222.50	47.53	373.00	99.00
50	114.27	112.67	28.76	212.67	47.67	475.18	473.50	45.33	598.00	360.33
60	317.29	316.67	33.62	403.33	235.00	807.27	808.33	46.25	925.00	673.00
70	623.29	623.33	43.72	708.67	512.33	1058.77	1059.50	48.16	1234.33	931.00
80	824.75	827.17	56.36	951.00	698.33	1268.80	1271.17	56.55	1465.33	1121.67
90	1075.15	1076.33	44.98	1170.33	968.67	1643.33	1641.67	60.23	1955.00	1507.33
100	1202.48	1195.67	32.03	1287.33	1147.33	1889.94	1889.00	28.65	1993.00	1824.67
